# Supplementary material for: Atrial Fibrillation Increases the Risk of Early-Onset Dementia in the General Population: Data from a Population-Based Cohort
Source: J Clin Med. 2020 Nov 14;9(11):3665. doi: 10.3390/jcm9113665 (PMC7697737; doi:10.3390/jcm9113665)

Supplementary information

Table S1. Definitions and ICD-10 codes used for defining the comorbidities and clinical outcomes.

	**Definitions**	**ICD-10 codes or conditions**
**Comorbidities**		
Hypertension	Defined from diagnosis* plus treatment	ICD-10: I10, I11, I12, I13, I15<br>Treatment: all kinds of blood pressure lowering medications
Diabetes mellitus	Defined from diagnosis* plus treatment	ICD-10: E10, E11, E12, E13, E14<br>Treatment: all kinds of oral antidiabetics and insulin.
Dyslipidemia	Defined from diagnosis*	ICD-10: E78
Heart failure	Defined from diagnosis*	ICD-10: I11.0, I50, I97.1
Peripheral arterial disease	Defined from diagnosis*	ICD-10: I70.0, I70.1, I70.2, I70.8, I70.9
Previous myocardial infarction	Defined from diagnosis*	ICD-10: I21, I22, I25.2
Chronic kidney disease	Defined from eGFR or diagnosis*<br>(if laboratory value was not available, diagnosis code was used)	eGFR <60mL/min per 1.73 m^2<br>ICD-10: N18, N19
ESRD	Defined from the national registry for severe illness.	Patients undergoing chronic dialysis or those who had received a kidney transplant.
Malignancy	Defined from diagnoses of cancer (non-benign)	ICD-10: C00-C97
COPD	Defined from diagnosis* plus treatment	ICD-10: J42, J43(except J43.0), J44<br>Treatment: inhaled corticosteroid, inhaled bronchodilators, or oral methylxanthine (>1 months).
Chronic liver disease	Defined from diagnosis of chronic liver disease, cirrhosis, and hepatitis	B18, K70, K71, K72, K73, K74, K76.1
**Clinical outcomes**		

Dementia	Defined from diagnosis	F00, F01, F02, F03, G30, G31<br>Prescription of dementia drugs<br>(rivastigmine, galantamine, memantine, or donepezil)
New onset atrial fibrillation	Defined from diagnosis* without previous insurance claim for AF	I48
**Exclusion Diagnosis**		
Previous ischemic stroke	Defined from diagnosis*	ICD-10: I63, I64
Previous TIA	Defined from diagnosis*	ICD-10: G45
Previous intracranial hemorrhage	Defined from diagnosis*	ICD-10: I60, I61, I62
Potential absence of non-valvular atrial fibrillation	Defined from any diagnoses of mitral stenosis or heart valve surgery	I05.0, I05.2, I34.2, Z95.2-4,<br>claim for valve replacement or valvuloplasty
Dementia	Defined from diagnosis	F00, F01, F02, F03, G30, G31<br>Prescription of dementia drugs<br>(rivastigmine, galantamine, memantine, or donepezil)

*To ensure accuracy, diagnosis was established based on one inpatient or two outpatient records of ICD-10 codes in the database.

Table S2. Baseline characteristics of overall population and propensity score (PS)-matched population.

	**Overall population**			**3:1 PS matching population**		
	**No AF (N=417,279)**	**Incident AF (N=10,983)**	**SMD**	**No AF (N = 30,920)**	**Incident AF (N=10,309)**	**SMD**
**Age, years**	55.5±9.1	61.7±9.9	0.651	61.7±9.8	61.7±9.9	-0.007
**Age > 65**	74,143 (17.8%)	4,531 (41.3%)	0.533	12,511 (40.5)	4250 (41.2)	0.016
**Female, %**	191,431 (45.9%)	4,318 (39.3%)	0.133	12,443 (40.2)	4,148 (40.2)	0.000
**BMI, kg/m^2**	24.0±2.9	24.3±3.1	0.101	24.3±3.1	24.3±3.1	-0.007
**SBP, mmHg**	125.8±16.6	130.2±17.6	0.255	130.3±17.4	130.1±17.6	-0.011
**DBP, mmHg**	78.4±10.7	80.1±11.0	0.154	80.1±10.8	80.0±11.0	-0.005
**Blood glucose, mg/dL**	98.4±26.8	101.9±32.5	0.118	101.9±30.5	101.9±32.9	0.002
**Total cholesterol, mg/dL**	198.9±36.9	195.4±38.0	-0.092	195.6±36.8	195.7±38.1	0.001
**Serum creatinine, mg/dL**	0.99 ±0.97	1.08±1.02	0.032	1.03±1.14	1.08±1.03	0.043
**Blood hemoglobin, mg/dL**	13.9±1.5	13.9±1.5	0.032	13.9±1.5	13.9±1.5	0.021
**Hypertension, n (%)**	94,332 (22.6%)	4,450 (40.5%)	0.393	12,569 (40.7)	4186 (40.6)	-0.001
**Diabetes, n (%)**	31,483 (7.5%)	1,359 (12.4%)	0.162	3,813 (12.3)	1,284 (12.5)	0.004
**Dyslipidemia, n (%)**	95,735 (22.9)	3,571 (32.5)	0.215	10056 (32.5)	3359 (32.6)	0.001
**Heart failure, n (%)**	10,191 (2.4%)	977 (8.9%)	0.282	2,555 (8.3)	917 (8.9)	0.023
**History of MI, n (%)**	3,423 (0.8%)	273 (2.5%)	0.131	548 (1.8)	261 (2.5)	0.052
**PAOD, n (%)**	7,150 (1.7%)	356 (3.2%)	0.098	894 (2.9)	330 (3.2)	0.018
**CKD or ESRD, n (%)**	2,764 (0.7%)	142 (1.3%)	0.064	405 (1.3)	136 (1.3)	0.001
**Osteoporosis, n (%)**	56,584 (13.6%)	2,042 (18.6%)	0.137	5,885 (19.0)	1,957 (19.0)	-0.001
**COPD, n (%)**	10,609 (2.5%)	708 (6.5%)	0.189	1,478 (4.8)	665 (6.5)	0.073
**History of liver disease, n (%)**	84,708 (20.3%)	2,828 (25.8%)	0.13	8,009 (25.9)	2,648 (25.7)	-0.005
**History of malignancy, n (%)**	27,063 (6.5%)	1,118 (10.2%)	0.134	3,129 (10.1)	1044 (10.1)	0.000

**CHA_2DS_2-VASc score**	1.0±1.0	1.6±1.3	0.43	1.56±1.32	1.58±1.33	0.016
**Income level**			0.044			0.025
Low	120,422 (28.9)	3,354 (30.5)		9,445 (30.6)	3138 (30.4)	
Middle	151,912 (36.4)	3,868 (35.2)		10856 (35.1)	3,624 (35.2)	
high	144,945 (34.7)	3,761 (34.2)		10619 (34.3)	3547 (34.4)	
**Smoking**			0.035			0.026
No	283,087 (71.7)	7,384 (71.4)		22133 (71.6)	7362 (71.4)	
Former	36,450 (9.2)	1,060 (10.3)		3174 (10.3)	1057 (10.3)	
Current	75,477 (19.1)	1,894 (18.3)		5613 (18.2)	1890 (18.3)	
**Alcohol consumption**			0.067			0.001
Low	308,731 (74.0)	8,000 (72.8)		22600 (73.1)	7557 (73.3)	
Moderate	67,780 (16.2)	1,610 (14.7)		4569 (14.8)	1509 (14.6)	
Heavy	40,768 (9.8)	1,373 (12.5)		3751 (12.1)	1243 (12.1)	
**Exercise**			0.167			0.117
None	7,882 (1.9)	153 (1.4)		466 (1.5)	151 (1.5)	
Seldom	10,850 (2.6)	158 (1.4)		485 (1.6)	153 (1.5)	
Regular	398,547 (95.5)	10,672 (97.2)		29969 (96.9)	10005 (97.1)	
**ACE inhibitor or ARB**	39,615 (9.5%)	1,982 (18.1%)	0.25	5,591 (18.1)	1,860 (18.0)	-0.001
**Beta-Blocker**	37,528 (9.0%)	2,015 (18.4%)	0.275	5,722 (18.5)	1896 (18.4)	-0.003
**Diuretic**	44,803 (10.4%)	2,294 (20.9%)	0.281	6,543 (21.2)	2150 (20.9)	-0.008
**K-sparing diuretics**	3,791 (0.9%)	305 (2.8%)	0.139	670 (2.2)	293 (2.8)	0.043
**NDHP-CCB**	3,744 (0.9%)	340 (3.1%)	0.158	606 (2.0)	312 (3.0)	0.068
**DHP-CCB**	53,735 (12.9%)	2,478 (22.6%)	0.256	7360 (23.8)	2330 (22.6)	-0.029
**Digoxin**	889 (0.2%)	249 (2.3%)	0.186	249 (0.8)	235 (2.3)	0.120
**Alpha-blocker**	8,574 (2.1%)	490 (4.5%)	0.136	1319 (4.3)	457 (4.4)	0.008
**Statin**	25,263 (6.1%)	1,018 (9.3%)	0.121	2,893 (9.4)	962 (9.3)	-0.001
**Antiarrhythmic agents**	139 (0.03%)	46 (0.42%)	0.081	23 (0.1)	42 (0.4)	0.068

**Aspirin**	36,385 (8.7%)	2,034 (18.5%)	0.289	5,595 (18.1)	1901 (18.4)	0.009
**P_2Y_{12} inhibitor**	2,020 (0.5%)	140 (1.3%)	0.085	377 (1.2)	131 (1.3)	0.005
**Anticoagulation**	179 (0.04%)	48 (0.44%)	0.081	24 (0.1)	45 (0.4)	0.071
**F/U duration, months, median (IQR)**	93 (84, 100)	96 (86, 101)	0.164	93 (83, 99)	96 (86, 101)	0.215

Values are expressed in No. (%), mean ± standard deviation (SD), or median (interquartile range; IQR). Differences between groups are expressed in systemic mean difference (SMD).

Abbreviation: AF, atrial fibrillation; BMI, body mass index; SBP, systolic blood pressure; DBP, diastolic blood pressure; MI, myocardial infarction; PAOD, peripheral artery occlusive disease; CKD, chronic kidney disease; ESRD, end-stage renal disease; COPD, chronic obstructive pulmonary disease; ACE, angiotensin-converting enzyme; ARB, angiotensin type II receptor blocker; NDHP, non-dihydropyridine; CCB, calcium channel blocker; DHP, dihydropyridine; F/U, follow-up**;** CHA_2DS_2-VASc score, (congestive heart failure, blood pressure consistently above 140/90 mm Hg or treated hypertension on medication, age ≥75 years, diabetes mellitus, prior stroke or transient ischemic attack or thromboembolism)–(vascular disease [e.g., peripheral artery disease, myocardial infarction, aortic plaque], age 65–74 years, female sex).

Table S3. Incidence of dementia during follow-up periods according to the AF status in the propensity score-matched population.

Dementia		Cases, No. (%)	Incidence*	Adjusted HR (95% CI)	
				Model I†	Model II‡
**Including Stroke**					
**Overall dementia**					
	No AF (n=30,920)	1,516 (4.9)	7	1.00 (Reference)	1.00 (Reference)
	AF (n=10,309)	828 (8.0)	11.3	1.90 (1.72-2.11)	1.89 (1.70-2.09)
**Age Subgroup**					
< 50	No AF (n=4,120)	3 (0.1)	0.1	1.00	1.00
	AF (n=1,487)	9 (0.6)	0.8	6.53 (1.96-21.81)	5.85 (1.60-21.39)
50-59	No AF (n=8,602)	53 (0.6)	0.8	1.00	1.00
	AF (n=2,743)	53 (1.9)	2.6	3.50 (2.28-5.38)	3.46 (2.24-5.35)
60-69	No AF (n=10,350)	369 (3.8)	5.4	1.00	1.00
	AF (n=3,399)	251 (7.4)	10.3	2.07 (1.72-2.50)	2.00 (1.66-2.41)
≥ 70	No AF (n=7,848)	1,064 (13.6)	21.2	1.00	1.00
	AF (n=2,680)	515 (19.2)	30.5	1.70 (1.49-1.94)	1.70 (1.49-1.94)
**Early-onset dementia**					
	No AF (n=29,473)	69 (0.2)	0.3	1.00	1.00
	AF (n=9,550)	69 (0.7)	1	3.97 (2.70-5.83)	3.99 (2.70-5.89)
**Late-onset dementia**					
	No AF (n=30,851)	1,447 (4.7)	6.6	1.00	1.00
	AF (n=10,240)	759 (7.4)	10.4	1.83 (1.64-2.03)	1.81 (1.63-2.02)
**Censored for Stroke**					
**Overall dementia**					
	No AF (n=30,9203)	1,367 (4.4)	6.4	1.00	1.00
	AF (n=10,309)	639 (6.2)	9.1	1.68 (1.49-1.89)	1.65 (1.46-1.86)

**Age Subgroup**					
< 50	No AF (n=4,120)	3 (0.1)	0.1	1.00	1.00
	AF (n=1,487)	6 (0.4)	0.5	2.91 (0.57-14.88)	2.12 (0.31-14.60)
50-59	No AF (n=8,602)	45 (0.5)	0.7	1.00	1.00
	AF (n=2,743)	39 (1.4)	2	2.84 (1.71-4.72)	2.73 (1.63-4.59)
60-69	No AF (n=10,350)	351 (3.4)	4.8	1.00	1.00
	AF (n=3,399)	189 (5.6)	8.1	1.87 (1.51-2.31)	1.80 (1.45-2.24)
≥ 70	No AF (n=7,848)	968 (12.3)	19.8	1	1
	AF (n=2,680)	405 (15.1)	25.6	1.51 (1.30-1.76)	1.50 (1.29-1.75)
**Early-onset dementia**					
No AF (n=29,473)		61 (0.2)	0.3	1.00	1.00
AF (n=9,550)		50 (0.5)	0.8	2.91 (1.82-4.63)	2.83 (1.77-4.54)
**Late-onset dementia**					
No AF (n=30,851)		1,306 (4.2)	6.1	1.00	1.00
AF (n=10,240)		589 (5.8)	8.4	1.64 (1.45-1.85)	1.61 (1.42-1.82)

Abbreviation: HR, hazard ratio; AF, atrial fibrillation

*Incidence: per 1000 person-year

†Model I was adjusted for age and sex.

‡Model II was additionally adjusted for hypertension, diabetes mellitus, dyslipidemia, heart failure, previous myocardial infarction, peripheral artery disease, chronic kidney disease, osteoporosis, chronic obstructive pulmonary disease, liver disease, malignant neoplasm, CHA2DS2-VASc score, cardiovascular medications (e.g. ACEi or ARB, beta-blocker, diuretics, statin, alpha-blocker, K-sparing diuretics, digoxin, calcium channel blocker, antiarrhythmic drug, aspirin, P2Y12 inhibitor, oral anticoagulants), economic status, alcohol consumption, smoking status, exercise habitus, follow-up duration, body mass index, systolic and diastolic blood pressure, blood glucose, total cholesterol and blood hemoglobin level.

Table S4. Types of dementia during follow-up periods according to the AF status in the propensity score-matched population.

	Cases, n (%)	Incidence*	Adjusted HR (95% CI)	
			Model I†	Model II‡
**Alzheimer Dementia**				
**Including Stroke**				
No AF (n=30,917)	1137 (3.7)	5.2	1.00 (Reference)	1.00 (Reference)
AF (n=10,308)	574 (5.6)	7.8	1.62 (1.42-1.83)	1.6 (1.41-1.82)
**Censored for stroke**				
No AF (n=30,917)	1049 (3.4)	4.9	1.00	1.00
AF (n=10,308)	464 (4.5)	6.6	1.47 (1.28-1.7)	1.45 (1.26-1.68)
**Vascular Dementia**				
**Including Stroke**				
No AF (n=30,917)	191 (0.6)	0.9	1.00	1.00
AF (n=10,308)	145 (1.4)	2	3.16 (2.48-4.02)	3.02 (2.37-3.86)
**Censored for stroke**				
No AF (n=30,917)	150 (0.5)	0.7	1.00	1.00
AF (n=10,308)	87 (0.8)	1.2	2.64 (1.95-3.57)	2.48 (1.82-3.37)

Abbreviation: HR, hazard ratio; AF, atrial fibrillation

*Incidence: per 1000 person-year

†Model I and ‡Model II were same as in the Supplementary Table 3.

Figure S1. Cumulative incidence of dementia before (A) and after (B) censoring for stroke in the propensity score-matched population.

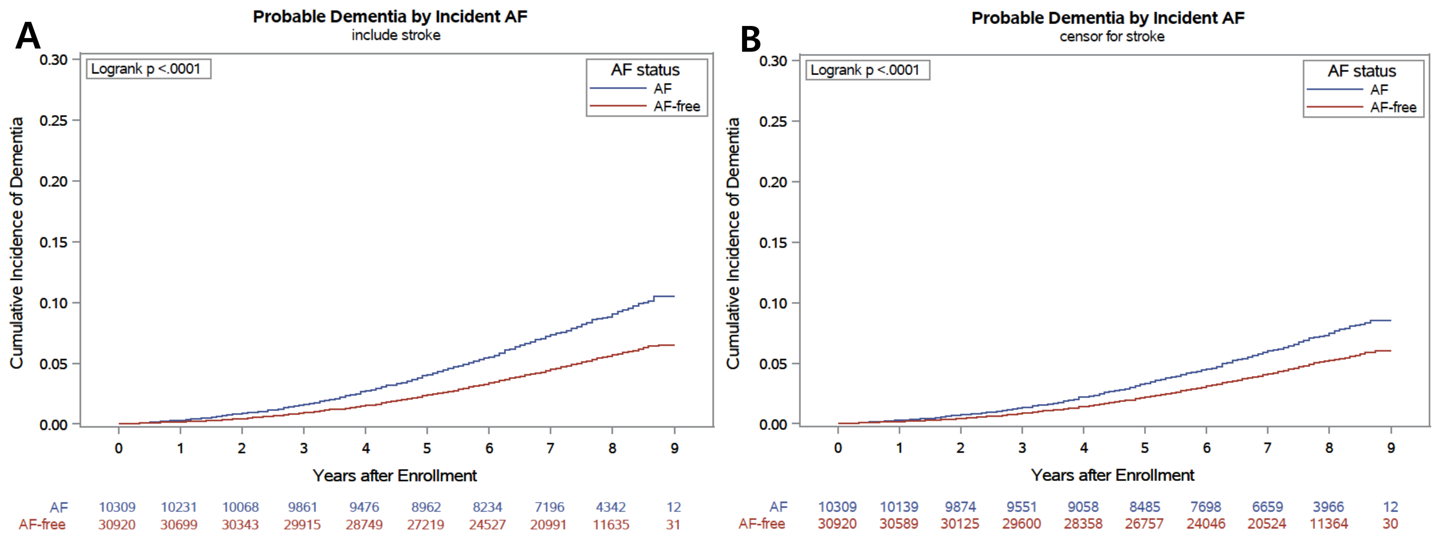

Figure S2. Hazard ratios for dementia per decade of age with atrial fibrillation in the propensity score-matched population.
(A) Including stroke during the follow-up. (B) Excluding stroke during the follow-up. Boxes indicate the hazard ratio, limit lines indicate the 95% confidence intervals, and the horizontal line (at hazard ratio 1) indicates no difference in hazard ratios between the atrial fibrillation (AF) and AF-free groups.

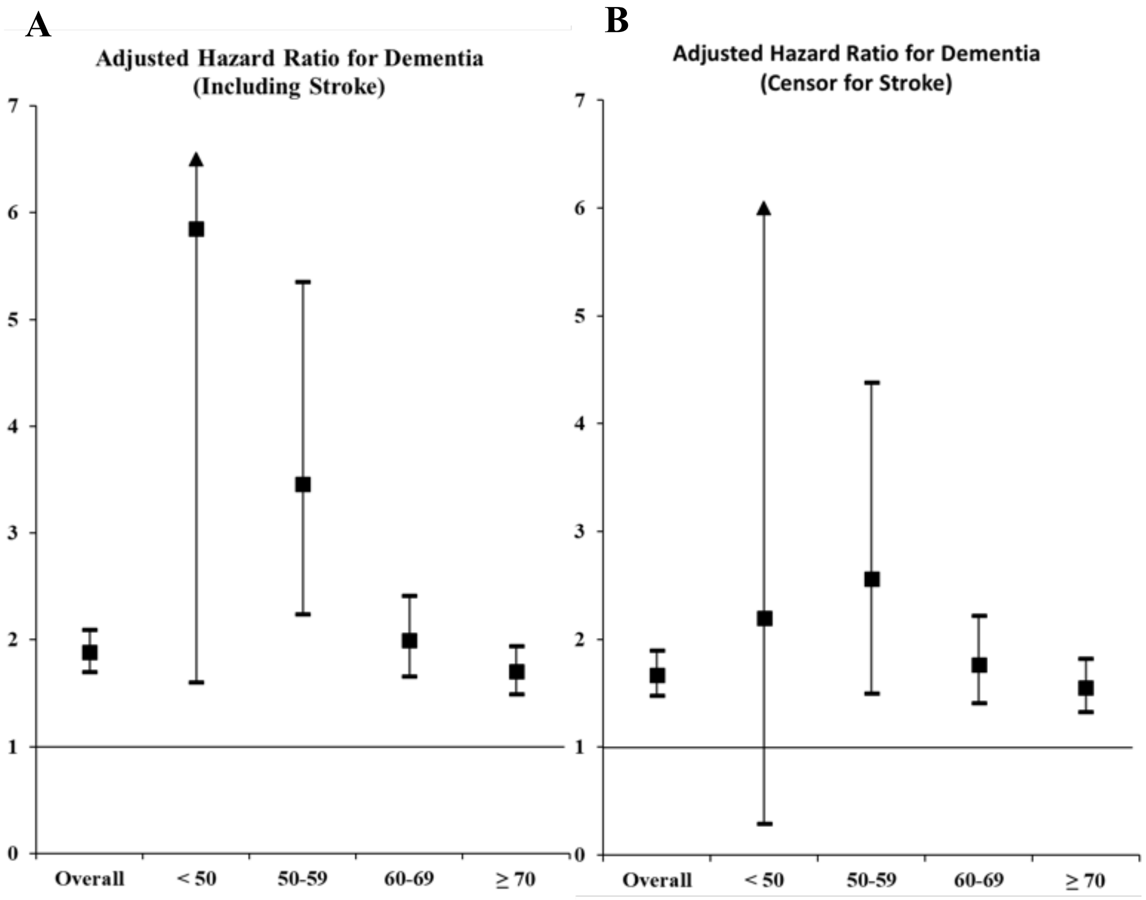

Supplement: Supplementary file 1 [file jcm-09-03665-s001.pdf]
